# Supplementary material for: Patterns of Intron Gain and Loss in Fungi
Source: PLoS Biol. 2004 Nov 30;2(12):e422. doi: 10.1371/journal.pbio.0020422 (PMC532390; doi:10.1371/journal.pbio.0020422)
Supplement: Table S1 — Also available at http://genes.mit.edu/NielsenEtAl/. (4.3 MB ZIP). [file pbio.0020422.st001.zip › NielsenEtAl/html/79.html]

AN7000.1.NCU08471.1.MG06977.1.FG10885.1


```
 CLUSTAL W (1.82) Multiple Sequence Alignments - Introns Inserted


Sequence 1: NCU08471.1	447 aa
Sequence 2: FG10885.1	445 aa
Sequence 3: MG06977.1	446 aa
Sequence 4: AN7000.1	456 aa
Alignment Length: 463 aa
Number Identitical Residues: 314 aa
Alignment Score (without introns) 13009


MG06977.1 	MFNVARNRVLASAIKTS~P~R~LPGLAR-PAFAQQQKRALSIHEYLSAELLSK0YGVQVP
NCU08471.1	MFKLGRNRALASAFAAT~S~R0APLASRLPSVSQQQRRALSIHEYLSADLLRQ0YGIGVP
FG10885.1 	MFKLGRSRAVASALNAS~K0F~VAPAARFPGV--QQRRALSIHEYLSADLLRQ0YGIGVP
AN7000.1  	MFTVLRSRPVAALRAAT0G~S~SSRIAQ-------QQRNLSIHEYLSANLLKS0YGVGVP
          	**.: *.* :*:   ::      .  ::       *:* *********:** . **: **

MG06977.1 	AGRVARSGEEAEQVAKEIG~HDDMVIKAQVLAGGRGKGTFDNGLKGGVRVIYS~PTEARM
NCU08471.1	KGDVARTGAEAEAIAKQIG~GEDMVIKAQVLAGGRGKGTFDNGLKGGVRVIYS~PTEAKM
FG10885.1 	QGSVAKSAKEAKEIAEKIG~NDDMVIKAQVLAGGRGKGTFDNGLKGGVRVIYS~PHEAEM
AN7000.1  	KGEVARSAEEAEKVAKSLG1NDDMVIKAQVLAGGRGKGHFDNGLKGGVRVIYS2PTEAKM
          	 * **::. **: :*:.:*  :**************** ************** * **.*

MG06977.1 	FADQMIGHKLITKQTGAQGRPCNAVYICERKFARREFYLAILMDRGSQSPVIVSSSQGGM
NCU08471.1	FAEQMIGHKLITKQTGAQGRLCSAVYICERKFARREFYLAVLMDRASQGPVIVSSSQGGM
FG10885.1 	FAEQMIGHKLVTKQTGAGGRLCNSVYICERKFARREFYLAILMDRQNQCPVIVSSSQGGM
AN7000.1  	FAGQMIGHKLITKQTGAGGRLCNAVFIVERKFARREFYLAVLMDRKTQGPVIVASSQGGM
          	** *******:****** ** *.:*:* ************:**** .* ****:******

MG06977.1 	DIEGVAKENPDAIVTTHIDINTGVTDETARDIAQKLGFSEQCIEDAKDTIQKLYKIFIEK
NCU08471.1	DIEGVAKENPEAIHTTYIDINVGVTDEMARDIATKLGFSEQCVEEAKDTIQKLYKIFCEK
FG10885.1 	DIEAVAKDTPDAINTNYIDINVGVTDETAREIATKLGFSEQCIEDAKDTIQKLYKIFREK
AN7000.1  	DIEAVAKENPDAIITTPIDINVGVTDAIATKIANELGFSEQCVDDAKKTIQNLYKAFMET
          	***.***:.*:** *. ****.****  * .** :*******:::**.***:*** * *.

MG06977.1 	DMTQIEINPLSETSDHKVMCMDAKFGFDDNAEFRQKEIFEKRDTTQEDAEEVRAAE~SGL
NCU08471.1	DATQIEINPLSETSDHKVMAMDAKFGFDDNADFRQPDIFKLRDTTQEDPDEVRAAQ~AGL
FG10885.1 	DSTQIEINPLSETSDHQVLCMDAKFGFDDNADFRQKEVFEWRDTTQEDPDEVRAAE~SNL
AN7000.1  	DATQIEINPLSETSDHQVLAMDAKLGFDDNAEFRQKEIFSWRDTTQEDADEVKAAE2HGL
          	* **************:*:.****:******:*** ::*. *******.:**:**:  .*

MG06977.1 	NFIKLDGDIGCL----------------VNGAGLAMATMDIIKLNGGSPANFLDVGGGAT
NCU08471.1	NFIKLDGDIGCL----------------VNGAGLAMATMDIIKLNGGQPANFLDVGGGAT
FG10885.1 	NFIKLDGDIGCL----------------VNGAGLAMATMDIIKLNGGQPANFLDVGGGAT
AN7000.1  	NFIKLDGDIGCLGVYPRPNDIIAVLTRTVNGAGLAMATMDIIKLNGGSPANFLDVGGGAT
          	************.  . ...  :  : :*******************.************

MG06977.1 	PAAIKEAFELITSDQKVSAVFVNIFGGIVRCDHIANGLINTVKTLNLKVPIIARLQGTNV
NCU08471.1	PAAIREAFELITSDPKVTAIFVNIFGGIVRCDAIAHGLINTVKSLDLKIPIIARLQGTNM
FG10885.1 	PAAIKEAFELITSDPKVTAIFVNIFGGIVRCDAIATGLIKTVESLNLKIPIIARLQGTNV
AN7000.1  	PAAIKSAFELITSDPKVSAIFVNIFGGIVRCDAIAQGLINVVQEMGLRTPIVARLQGTNM
          	****:.******** **:*:************ ** ***:.*: :.*: **:*******:

MG06977.1 	EQAHQI~INDSGMKIFSIDDLQSAAEKAVQLSKVVKMA1RDIDVGVEFSLGI
NCU08471.1	EAARQL~INDSGMKIFSIDDLQSAAEKSVQLSKVVKMA1RDIDVGVEFTLGI
FG10885.1 	EKAHQL~INDSGLKIFSIDDLQTAAEKSVQLSKVVKMA1RDIDVGVEFTLGI
AN7000.1  	EQAHKL0INESGLKIFSIEDLQSAAEKSVQFSKVVKMA~REIDVGVEFTLGI
          	* *::: **:**:*****:***:****:**:******* *:*******:***
```
